# Stuck between Bench and Bedside: Why Non-invasive Brain Stimulation Is Not Accessible to Depressed Patients in Europe

**DOI:** 10.3389/fnhum.2017.00039

**Published:** 2017-02-03

**Authors:** Anna-Katharine Brem, Soili M. Lehto

**Affiliations:** ^1^Max Planck Institute of PsychiatryMunich, Germany; ^2^Department of Experimental Psychology, University of OxfordOxford, UK; ^3^Berenson-Allen Center for Noninvasive Brain Stimulation, Division of Interventional Cognitive Neurology, Department of Neurology, Beth Israel Deaconess Medical Center, Harvard Medical SchoolBoston, MA, USA; ^4^Department of Psychology, Institute of Behavioral Sciences, University of HelsinkiHelsinki, Finland; ^5^Department of Psychiatry, Institute of Clinical Medicine, University of Eastern FinlandKuopio, Finland; ^6^Department of Psychiatry, Kuopio University HospitalKuopio, Finland

**Keywords:** depression, transcranial magnetic stimulation, transcranial electrical stimulation, transcranial direct current stimulation, guidelines

Major depression is the leading psychiatric disorder in developed countries, and the leading cause of disability and burden of disease worldwide, with 350 million people currently being affected (WHO, 2014). While treatment in the last decade has improved for stroke, cardiovascular disease, and cancer, there has been little improvement in the pharmacological treatment of depression. A majority of research funding is invested in the examination of pharmacological agents despite known limitations of their efficacy (Penn and Tracy, 2012; Wilkinson and Izmeth, 2016). Current treatment culture appears to be biased toward pharmacological treatment, possibly due to effective marketing and ease of use for both clinicians and patients, while other forms of treatment, albeit promising, receive less attention. The advancement of alternative treatment modalities is specifically relevant to patient groups that are known to be vulnerable to the side effects of antidepressants, e.g., the rapidly growing population of older adults with late-life depression (Copeland et al., 2004; Vaughan et al., 2015) and pregnant and lactating women (Pearlstein, 2008).

The efficacy of transcranial magnetic stimulation (TMS) for the treatment of depression has been demonstrated in numerous studies (Lefaucheur et al., 2014), while less evidence exists as to the efficacy of transcranial electrical stimulation (tES), especially transcranial direct current stimulation (tDCS) (Meron et al., 2015; Brunoni et al., 2016; Lefaucheur et al., 2017). The National Health and Care Excellence (NICE, UK), in its interventional guidance procedure, updated at the end of 2015 and well regarded by other European countries, recommends TMS as a first-line treatment and remarks on its good safety profile. Conversely, due to the inconsistency of results to date, NICE recommends tDCS to be used only with special arrangement in the context of clinical practice (www.nice.org.uk/guidance) and encourages further research.

An efficient and smooth translation of cutting-edge research findings into clinical applications with the final end point of accessibility for patients requires a fast transition of knowledge, is characterized by successful collaboration between clinicians, researchers, and policy makers, and requires continuing refinement within and across each stage. This process involves the development and improvement of intervention methods, testing in animal models and humans, and careful development of clinical trials, before policy makers evaluate the given evidence and release treatment guidelines. As the last and most important step for patient accessibility, national health insurance entities decide on the integration of a given method in their armamentarium of treatment approaches. Although, it seems evident that an efficient translation from bench to bedside of novel treatment methods, such as non-invasive brain stimulation (NIBS), is imperative in the fight against depression, such advancements are proceeding at a very leisurely pace. However, besides this rather well-defined trajectory there are other factors that account for the final accessibility of novel treatment methods for patients, of which various appear to hinder effective translation: Residency programs in psychiatry offer only limited training in brain stimulation, resulting in insufficient knowledge and awareness. In addition, lack of infrastructure and resources in many hospitals prevents the organization of an efficient treatment system. Finally, treatment costs remain subject to differential health insurance policies (Lefaucheur et al., 2014). Currently, several non-European countries (USA, Canada, Brazil, Australia, Israel) approve the use of TMS as a first- or second-line treatment in depression, while few European (Germany, Finland, Serbia) countries reimburse the costs of TMS treatment (Lefaucheur et al., 2014). The limited translation of knowledge from bench to bedside gives rise to a number of ethical issues. Firstly, patients are being forced to turn to the growing number of private practices that offer off-label treatment by therapists who may lack certified training. Secondly, the inadequate availability of NIBS treatments may encourage seeking help from internet-based communities, and promote the home-use of unapproved tES devices and protocols (Wurzman et al., 2016). Thirdly, patients who are especially vulnerable to side effects of pharmacological antidepressant treatments continue to receive a non-optimal treatment, leading to increased individual suffering and rising costs for society. In the case of pregnant women, negative effects may also lead to further adverse effects. For example, exposure to selective serotonin reuptake inhibitors (SSRI) during pregnancy has been associated with cognitive impairment in the offspring (Brown et al., 2016). In older adults, efficacy of pharmacological treatment is reduced (Wilkinson and Izmeth, 2016) and interactions with other medication, as well as the physiological strain of multimedication, pose an additional risk. In late-life depression, a condition associated with an increased risk for dementia, even a small improvement in depressive symptoms may lead to a large advancement associated with secondary personal, familial, and social benefits.

The above shortcomings and ethical issues require explicit consideration when creating policies for the use of NIBS in the treatment of depression. Factors responsible for slow translation from research to clinical practice need to be analyzed and addressed. Effective translation from bench to bedside with the aim of increasing accessibility for patients continues to require strong, ongoing support for research seeking to enhance knowledge of the most effective way for utilizing NIBS in treatment of depression. Translational efforts should be encouraged via EU funding schemes specifically dedicated to non-pharmacological research in depression (e.g., industry-academy collaborations), the investigation of underlying mechanisms and individual markers associated with treatment success, as well as long-term follow-up of effectiveness. Moreover, novel tES protocols such as transcranial alternating current stimulation (tACS) and transcranial random noise stimulation (tRNS), a special form of tACS (Antal and Herrmann, 2016) as well as rTMS protocols such as accelerated rTMS (McGirr et al., 2015) and theta-burst stimulation (Chung et al., 2015) merit in-depth investigation.

The development of certified home-use devices, which is specifically relevant for tES as it bears less risk than rTMS, would further reduce costs and allow investigating larger patient groups. However, this approach also requires special attention with regards to misuse. Instead of approving devices (such as Neuronetics in the U.S.A.), the approval of specific protocols should be promoted and published in international guidelines. This will lead to a market-driven cost reduction, incite health insurances, and national health systems to include NIBS as a treatment option, and consequently improve access to NIBS treatments. More research in vulnerable populations, particularly older adults and pregnant/lactating women, and the explicit acknowledgment of these groups in treatment guidelines is warranted. Furthermore, training standards and guidelines need to be defined on a pan-European level (Lefaucheur et al., 2017). Only adequately trained health specialists should be allowed to offer NIBS treatment and training in delivering NIBS interventions. These interventions should be available not only through neurophysiologists but also through psychologists and psychiatrists and constitute an integral part of their general training requirements and ongoing education. Implementation of active training approaches will consequently lead to improvements in infrastructure and resources. Thus, joint educational and research input from European universities is needed to facilitate the effective development and application of NIBS methods in treatment of depression.

## Author contributions

AB and SL drafted and revised the manuscript.

### Conflict of interest statement

The authors declare that the research was conducted in the absence of any commercial or financial relationships that could be construed as a potential conflict of interest.
